# From IOTA Tangle 2.0 to Rebased: A Comparative Analysis of Decentralization, Scalability, and Suitability for IoT Applications

**DOI:** 10.3390/s25113408

**Published:** 2025-05-28

**Authors:** Pierre Sedi Nzakuna, Vincenzo Paciello, Aimé Lay-Ekuakille, Angelo Kuti Lusala, Salvatore Dello Iacono, Antonio Pietrosanto

**Affiliations:** 1 Department of Industrial Engineering, University of Salerno, 84084 Fisciano, Italy; 2 Department of Innovation Engineering, University of Salento, 73100 Lecce, Italy; 3 Department of Electronics, Institut Supérieur Pédagogique Technique de Kinshasa, Kinshasa, Democratic Republic of the Congo; 4 Department of Electrical and Computer Engineering, Polytechnic Faculty, University of Kinshasa, Kinshasa XI BP 127, Democratic Republic of the Congo; 5 Department of Information Engineering, University of Brescia, 25121 Brescia, Italy

**Keywords:** Internet of Things, IOTA, distributed ledger technologies, blockchain, directed acyclic graph

## Abstract

The Internet of Things (IoT) demands scalable, secure, and feeless distributed ledger technologies (DLTs) to enable seamless machine-to-machine transactions. The IOTA DLT was developed to fulfill this vision through its feeless Directed Acyclic Graph (DAG) named the Tangle, whose announced upgrade to IOTA 2.0 promised feeless microtransactions and coordinator-free (Coordicide) decentralization via a Nakamoto Consensus mechanism and a Mana anti-spam system. However, its delayed decentralization and scalability limitations hindered ecosystem growth and practical IoT adoption, leading to a new ledger architecture named IOTA Rebased. This paper critically analyzes this architectural pivot and its implications for IoT applications, contrasting the abandoned IOTA 2.0 protocol—a leaderless, feeless DAG designed for the IoT—with the adoption of a Move Virtual Machine-based, object-oriented ledger secured by a Delegated Proof-of-Stake consensus via the Mysticeti protocol in IOTA Rebased. We evaluate IOTA Rebased trade-offs: enhanced programmability and speed versus compromised IoT suitability due to fees, and explore mitigation strategies such as sponsored transactions, lightweight clients, and hierarchical tiered transaction architecture to align IOTA Rebased with IoT environments where microtransactions are prevalent. A use case analysis is provided for the integration of IOTA Rebased in IoT scenarios. This study underscores the tension between technological innovation and decentralization, offering insights for balancing scalability with the unique demands of the IoT.

## 1. Introduction

The Internet of Things (IoT) refers to a vast network of interconnected physical objects—ranging from everyday household items to sophisticated industrial sensors—each equipped with unique identifiers that enable autonomous data exchange [1]. As these devices proliferate, ensuring secure, scalable, and efficient communication becomes increasingly challenging, particularly due to centralized vulnerabilities and resource constraints inherent in traditional architectures [2]. Distributed Ledger Technologies (DLTs) emerge as a transformative solution in this context by facilitating decentralized, tamper-proof, and transparent data management. DLTs provide robust mechanisms for ensuring data integrity and trust through immutable records and consensus protocols that eliminate single points of failure in the network [1]. Adaptive consensus techniques within DLTs can achieve fairness among heterogeneous devices, making them particularly suited for the high-frequency, low-value transactions typical of IoT applications [3].

IOTA emerged in this context as an open-source, feeless DLT specifically designed to meet the demands of IoT applications, offering high scalability through its innovative Directed Acyclic Graph (DAG) structure named the Tangle [4]. The DAG structure of the Tangle, contrasting with the traditional blockchain structure, allowed IOTA to process a higher volume of microtransactions with zero fees, thus achieving high scalability since all nodes in the network acted as validators by validating existing transactions in the network before inserting their own. Additionally, IOTA implemented a lightweight Proof-of-Work (PoW) mechanism well suited for the low-power requirements of IoT hardware, thereby eliminating the cost barrier for IoT devices with limited computational resources. This approach made IOTA a promising solution to address the decentralized, scalable, and secure communication needs of the IoT and machine-to-machine transactions [5,6].

However, the IOTA Tangle faced the blockchains trilemma that they cannot be decentralized, scalable, and secure at the same time. The Coordinator was then introduced in the IOTA Tangle as a temporary bootstrapping mechanism to enhance security and finality by issuing milestones to reach consensus. This solution came at the cost of introducing a centralized element into what was inherently meant to be a decentralized system, thereby creating a potential single point of failure in the network [2]. To address this contradiction, IOTA 2.0 was designed with the goal of executing “Coordicide”, an update aimed at removing the coordinator from the network, to allow full decentralization while preserving the scalability and security that are critical for the IoT environment [7,8]. In addition, IOTA 2.0 aimed at introducing a novel Nakamoto Consensus mechanism with a reputation system known as Mana and a sharding-inspired approach for scalability to facilitate seamless, feeless microtransactions across billions of connected devices.

Despite the groundbreaking vision of IOTA 2.0, practical issues and changing network demands prevented its deployment. In response, the IOTA Foundation has shifted its focus toward a reimagined protocol known as IOTA Rebased [9], which departs significantly from its predecessors by incorporating transaction fees to sustain network operations and leverages the Mysticeti DAG structure [10] in replacement of the Tangle to bolster throughput and security. While these changes enhance certain aspects of network performance and resilience, they also introduce complexities, particularly the reintroduction of fees, which may affect the feasibility of microtransactions that are critical to IoT applications.

This paper provides a systematic comparative analysis of the architectural evolution of IOTA, grounded in a structured framework that contrasts the decentralized, feeless promises of IOTA 2.0 with the fee-driven realities of IOTA Rebased. We derive evaluation criteria from IoT requirements—scalability, microtransaction feasibility, decentralization, and hardware efficiency—to assess trade-offs between these protocols and reveal how the shift from a leaderless DAG to a staking-driven object-oriented ledger impacts IoT adoption. Our analysis synthesizes insights from peer-reviewed case studies and protocol whitepapers while aligning findings with established DLT literature. In addition, this work proposes actionable mitigation strategies, such as sponsored transactions and tiered architectures, to reconcile enhanced programmability in Rebased with IoT microtransaction demands. Furthermore, we critically evaluate the consequences of abandoning feeless transactions, a cornerstone of the original vision of IOTA, on resource-constrained IoT ecosystems. By dissecting the strengths and limitations of both protocols through this rigorous comparative analysis, this study aims to equip researchers and practitioners with insights to bridge the gap between IoT-friendly decentralization and modern ledger governance.

The remainder of the paper is structured as follows: Section 2 summarizes the evolution of the IOTA Tangle. Section 3 reviews the promises and technical foundations of IOTA 2.0. Section 4 explains the new IOTA Rebased architecture, including its consensus mechanism and tokenomics. Section 5 presents a comparative analysis of IOTA 2.0 with IOTA Rebased, especially in the context of IoT applications. Section 6 proposes recommendations for the integration of IOTA Rebased in the IoT—according to its original mission of enabling efficient and cost-effective machine-to-machine transactions—with a use case analysis. Section 7 proposes potential future research directions, and finally, the last Section 8 concludes this work.

## 2. Evolution of the IOTA Tangle

### 2.1. The Tangle

**Figure 1:**
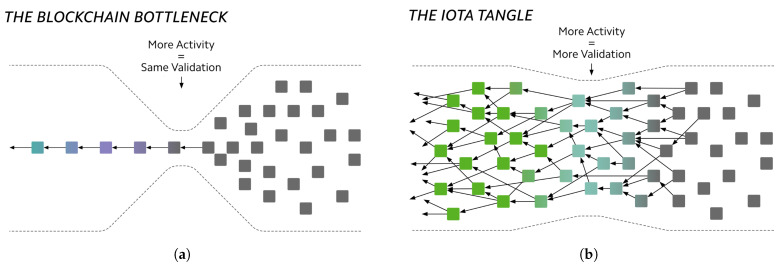
The DAG structure of the Tangle solves the scalability issue in blockchains expressed by the bottleneck. Black squares represents transactions waiting to be added in the ledger. (**a**) The single-chain structure of blockchains, where only one new transaction can be added to the ledger at a time. (**b**) The DAG structure of the IOTA Tangle, allowing multiple transactions to be simultaneously added to the ledger. Green squares represents finally-confirmed transactions.

The DAG structure of the Tangle overcomes the scalability limitations of traditional blockchains by allowing each transaction to reference multiple predecessors simultaneously, instead of only one as in blockchains. This design in the Tangle enabled several transactions to be attached and validated concurrently, thus significantly enhancing the network throughput and efficiency [11]. Figure 1 shows the difference between the DAG structure of the Tangle and the single-chain structure of blockchains, with the Tangle solving the bottleneck and throughput issues in blockchains.

Vertices in the Tangle DAG are called blocks, and edges are called *references*. A new block in the Tangle is known as a tip (dark gray blocks on Figure 1b). When first issued, a tip is unconfirmed and must reference at least two earlier blocks to validate them. As additional blocks are issued and reference the tip, peer nodes in the network gradually increase its confirmation confidence. The more a tip is referenced by subsequent blocks, the more reliable it becomes, thereby raising its likelihood of reaching final confirmation [12]. The computational effort required to validate a block determines its weight. The cumulative weight of a block is calculated by adding its own weight to those of all transactions that directly or indirectly approve it, which increases its significance within the ledger. Consequently, a tip remains unconfirmed until its cumulative weight reaches a specified threshold, signifying that it has garnered sufficient network validation [2].

When issuing a new tip in the network, a node selects the two blocks to reference using a weighted Markov Chain Monte Carlo random walk algorithm. This algorithm employs a regulating parameter α, which prevents the selection process from always favoring the tip with the highest cumulative weight, thereby ensuring a more balanced and secure tip selection [12]. Consensus within the Tangle is achieved when nodes collectively agree on which blocks should be included and validated in the ledger, with the cumulative weight of a block serving as a crucial indicator of its legitimacy [2]. The Tangle relies on a Proof-of-Authority (PoA) consensus mechanism—leveraging the verifiable identity of each node as a form of stake—along with a lightweight PoW that must be performed for every transaction. This combination not only helps establish trust in the network but also protects it from flooding attacks by malicious entities attempting to overwhelm the system with spurious blocks [11].

The Coordinator—a node plugin controlled by the IOTA Foundation—functions as a centralized finality mechanism by periodically issuing signed blocks called milestones. These milestones are trusted by other nodes in the network and serve as confirmation checkpoints, so that any block in the Tangle that is either directly or indirectly referenced by a milestone is deemed confirmed [13]. However, this centralized approach is controversial within the cryptocurrency community because it grants the IOTA Foundation the authority to effectively decide which transactions receive priority, potentially freeze funds, or even ignore certain transactions. Moreover, the reliance on the coordinator creates a single point of failure because if it were to stop functioning, the entire confirmation process in the network would come to a halt [2].

Network roles are divided among full nodes, perma nodes, and clients. Nodes (both full and perma) use a consensus algorithm to validate transactions. Perma nodes hold the entire ledger history, while full nodes focus on the current ledger state. To send transactions, clients must connect to the ledger via either a full or perma node. The Tangle network ecosystem includes the Mainnet (production network), Shimmer (staging network for pre-Mainnet protocol testing), and the public testnet (developer test bed) [13].

Unlike classical modeling tools such as finite-state automata and Petri nets, which routinely use cycles to represent recurring control flows and resource replenishment, the Tangle is built on an enforced acyclicity guaranteeing that once a transaction is confirmed, it cannot be revisited, thereby eliminating infinite-loop scenarios and delivering deterministic, irreversible settlement. While Petri nets model concurrency through tokens and synchronizing transitions inside potentially cyclic structures [14], the Tangle captures concurrency by recording the causal order between independent transactions, allowing many tips to be approved in parallel. By trading the expressiveness of cycles for a growing, one-way DAG, the Tangle achieves the high throughput and rapid finality demanded by large-scale ledger applications.

### 2.2. Network Protocol Updates

The IOTA Tangle has evolved through four major protocol iterations since its initial release in 2016. The initial version, later on known as IOTA 1.0, employed a trinary data representation coupled with a probabilistic consensus mechanism based on random walkers that navigated toward the tips to select candidate transactions for approval [4]. This early design, however, was particularly susceptible to conflict spamming attacks. In response, the first Chrysalis protocol update was introduced, upgrading the ledger to the IOTA 1.5 version, and mitigating these vulnerabilities by ignoring conflicting transactions during tip selection. By imposing a deterministic order on the Tangle, the Chrysalis update enabled the use of milestones to confirm transactions by accepting only the first non-conflicting message that maintained the integrity of the ledger.

The second Chrysalis upgrade marked a pivotal transformation for IOTA by shifting the data representation of the ledger from trinary to binary, thereby streamlining data processing and interoperability with modern systems. The operating model shifted from the traditional account model to an Unspent Transaction Output (UTXO) framework, allowing for more precise and conflict-resistant tracking of funds. Additionally, the upgrade replaced the Winternitz One-Time Signature scheme with the more robust Edwards-curve Digital Signature Algorithm (EdDSA), thereby enhancing both security and computational efficiency considering IoT devices with limited resources. Protective measures were also introduced for microtransactions by ensuring recipient addresses could resist dust attacks (wherein negligible amounts of funds are sent to clutter and potentially exploit the network) [13].

The Stardust upgrade introduced the support of multiple assets beyond the base IOTA token. This update refined the dust protection in the ledger by introducing a more granular protection mechanism based on precise byte cost calculations through storage deposits. Furthermore, Stardust rebranded the basic unit of the ledger from messages to blocks, reflecting a maturation of the underlying architecture. It also laid the foundation for IOTA Smart Contracts (ISCs) by incorporating Alias Outputs for efficient anchoring of smart contracts by guaranteeing there is always exactly one such output for each chain of the UTXO ledger [15], and adopted the Bench32 format for address encoding, which provides strong error correction. The single coordinator node was replaced with a distributed validator committee using an off-chain Byzantine Fault Tolerant (BFT) consensus mechanism, a leap towards the full decentralization goal of IOTA. Table 1 summarizes the four IOTA Tangle protocol versions and their main characteristics.

The Shimmer Ethereum Virtual Machine (EVM) was released along with the Stardust protocol to support ISCs, allowing developers to leverage existing Ethereum smart contracts and tools on the Shimmer EVM chain while demonstrating noticeable performance, such as high Transactions Per Second (TPS) and low latency [16]. The Shimmer EVM integrated the support of Layer 1 native assets and Non-Fungible Tokens (NFTs), with the aim of allowing the creation and operation of multiple individual Layer 2 networks running in parallel in future upgrades [17]. Following the release of the Shimmer EVM, ISCs underwent extensive improvements, leading to the release of IOTA EVM by June 2024, a Layer 2 solution designed to bring smart contract capabilities to the IOTA network. IOTA EVM ensures full EVM compatibility and adds innovative features leveraging the unique Layer 1 Native Asset Framework of IOTA. IOTA EVM leverages its unique Layer 1 for parallel processing, enabling horizontal scalability and boosting multi-chain performance. It offers seamless interoperability via easy Solidity contract deployment across EVM and non-EVM chains. Furthermore, native randomness and resistance against Maximal Extractable Value (MEV) enhance fairness and security by preventing front-running through unpredictable transaction ordering, promoting market integrity [18].

## 3. IOTA 2.0: Promises and Challenges

### 3.1. Core Concepts

IOTA 2.0 was conceived as a paradigm shift from the coordinator-dependent architecture of its predecessors, aiming to achieve full decentralization while preserving scalability and security for IoT applications. This subsection delineates the foundational pillars of the IOTA 2.0 design, which sought to eliminate centralized oversight through leaderless consensus, dynamic resource allocation, and cryptoeconomic incentives.

#### 3.1.1. Leaderless Full Decentralization

The flagship objective of IOTA 2.0 was to remove the centralized coordinator from the network. The removal of this single point of control—and potentially of failure—, referred to as Coordicide, was intended to pave the way for a trustless and fully decentralized network. In the absence of the coordinator, every network participant would contribute to the validation process, thus performing autonomous transaction validation in the ledger. This approach was designed to enhance network resilience by distributing trust and security across nodes [19].

**Figure 2:**
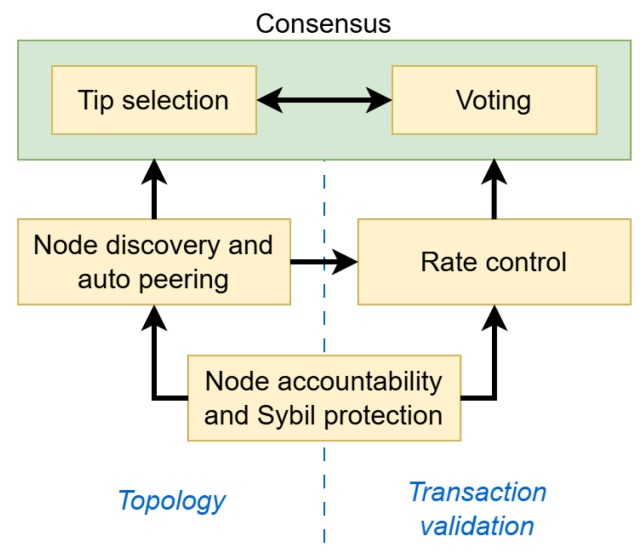
Building blocks in the Coordicide architecture [7].

Various challenges needed to be tackled to achieve effective removal of the coordinator, summarized as building blocks in Figure 2. Firstly, node accountability was implemented through unique global node IDs, combined with an innovative Sybil protection mechanism that avoided requiring operators to risk or reveal funds. By reliably identifying which node sent a message, the network could automatically organize itself through auto peering and effectively discourage harmful actions by implementing measures like rate control against malicious nodes. Secondly, a node discovery and auto-peering mechanism was proposed to facilitate easy network entry for new nodes while also securing the peering process against targeted attacks, thereby protecting the network from Eclipse attacks. Thirdly, a rate control mechanism was designed to prevent congestion by selectively allowing transactions to propagate based on statistics related to the originating node. To issue transactions, the latter had to solve a cryptographic puzzle to issue a transaction whose difficulty dynamically scaled, increasing with recent transaction frequency and decreasing with the Mana owned by that node, thus regulating throughput. Finally, to achieve consensus, two voting mechanisms were proposed to allow nodes to determine the current network status by querying the opinions of other nodes [7].

#### 3.1.2. Consensus Mechanism

While previous network protocol versions relied on the centralized coordinator and the weighted Markov Chain Monte Carlo to achieve consensus to finally confirm objects in the ledger, IOTA 2.0 initially relied on a combination of Fast Confirmation of On-the-Heavy Branch (FCOB) and Fast Probabilistic Consensus (FPC) as a probabilistic leaderless binary voting protocol, lightweight and scalable to provide rapid and energy-efficient consensus [19]. Instead of relying on resource-intensive mining, FPC leverages random sampling and iterative voting to quickly achieve agreement among network participants, with this procedure being terminated locally if a node has not changed its opinion over a specified period of time or if some maximal amount of rounds is reached [20]. The probabilistic nature of the consensus not only boosted efficiency but also added a layer of unpredictability that helps protect the network from coordinated attacks, thereby implementing security through randomness by ensuring that no single entity could easily manipulate the validation process. The voting power in FPC is proportional to the node reputations, and several improvements were implemented to decrease the failure rates significantly and allow IOTA to withstand adversaries with higher weight [21].

However, while FPC offers advantages in terms of simplicity and resistance to certain attacks, it was primarily effective for conflict resolution and did not provide a comprehensive solution for achieving global consensus across the network. In addition, the voting process performed via direct queries between the peers required an additional communication layer. Recognizing the limitations of FPC, the IOTA Foundation transitioned to a more robust consensus model inspired by the Nakamoto Consensus, adapting the traditional longest-chain rule from blockchain systems to the Tangle DAG structure [22,23]. In this model, validators, selected through a staking mechanism, issue specialized validation blocks at regular intervals. These validation blocks carry the validator stake weight and contribute to the approval weight of referenced blocks. The approval weight determines the consensus status of transactions and blocks, guiding the network toward a unified ledger state. This approach enables an asynchronous, leaderless, and scalable consensus process, allowing for parallel transaction validations without the need for a global total order [23].

To manage conflicting transactions and ensure network coherence in this adaptation of the Nakamoto Consensus, the protocol employs consensus flags and slot commitment chains. Consensus flags indicate the confidence level in the validity of a block, ranging from pre-acceptance to finalization. Slot commitment chains allow nodes to track and compare their views of the ledger, facilitating agreement on the most valid chain (with the heaviest cumulative weight). In cases of divergence, nodes adopt the chain with the highest cumulative weight, ensuring convergence to a single, consistent ledger state [23].

The Distributed Random Number Generation (dRNG) mechanism—inspired by voting-based Byzantine consensus protocols [24]—was added to address synchronization and prevent adversarial exploits by resolving scenarios where nodes might otherwise remain indecisive due to conflicting valid opinions. By generating synchronized external randomness at predetermined epochs, the protocol periodically aligns the state across nodes, breaking potential stalemates and facilitating rapid convergence towards consensus. This reduces vulnerabilities to adversarial tactics aimed at prolonging indecision within the network, ensuring more robust and predictable consensus outcomes [22].

#### 3.1.3. Reputation and Resource Management

To incentivize honest participation, IOTA 2.0 proposed a reputation system based on Mana. This mechanism assigns a reputation score to nodes based on their historical behavior and the volume of legitimate transactions they process, thereby acting as a critical resource governing access to network resources, securing the ledger, and ensuring fairness without the need for traditional transaction fees [20]. Mana was intended to ensure that nodes with higher reputation and investment in the network would have a more significant influence in the consensus process. This design was critical in mitigating Sybil attacks and maintaining overall network integrity.

In IOTA 2.0, Mana is accrued by holding IOTA tokens, with its amount dependent on both token quantity and holding duration. Mana serves the two following main functions:

- Stored Mana: Linked to token outputs and transferable, this type of Mana gradually decays over time to encourage continuous participation.
- Block Issuance Credits (BIC): Non-transferable credits enabling users to issue blocks, with usage dynamically adjusted based on network congestion, deterring spam and misuse.

Additionally, users can stake tokens to act as validators, directly contributing to consensus and network stability, or delegate tokens to trusted validators. Validators and delegators earn rewards based on their network contributions and performance, incentivizing reliable participation. To further avoid centralization, Mana and delegation rewards diminish as more tokens concentrate with a single validator, promoting equitable distribution and active engagement across the network [25].

#### 3.1.4. Smart Contracts

IOTA 2.0 featured ISCs, a scalable multi-chain smart contract framework anchored to the main ledger (Layer 1). ISC was proposed to support multiple parallel programmable ledgers (Layer 2 chains), each leveraging the UTXO model and quasi-Turing complete programmability to enhance scalability and efficiency. Cross-chain interactions occurred trustlessly via Layer 1 anchors, preserving decentralization and security. Additionally, ISC employed advanced cryptographic techniques such as threshold signatures and Verkle trees for secure state validation and efficient transaction processing, enabling practical, high-throughput smart contract applications [15].

#### 3.1.5. Security Enhancements

IOTA 2.0 incorporated advanced cryptographic techniques to secure transactions and protect against fraudulent activities. The decentralized validation process, combined with the FPC consensus and Mana-based reputation, was expected to offer a high level of security without sacrificing speed. By distributing both the validation responsibility and the control of network resources, IOTA 2.0 aimed to build a system that was inherently resistant to many conventional attack vectors found in centralized or less DLTs, such as the consequences of denial-of-service (DoS) attacks [26].

Beyond ledger-specific cryptography, the security posture of any IoT-centric DLT must be viewed in the wider cyber-physical-systems (CPS) threat landscape. Comprehensive surveys [27,28] catalogue attack types (sensor spoofing, control-loop hijacking, eavesdropping, etc.) and defense measures (defense-in-depth, data cleaning, elastic model predictive control, etc.) that span both cyber and physical domains. The layered security model proposed for IOTA 2.0—combining lightweight PoW, reputation-weighted rate control, and slot-anchored finality—aligned with the multi-tier mitigation strategies highlighted in these surveys.

### 3.2. Protocol Layers

**Figure 3:**
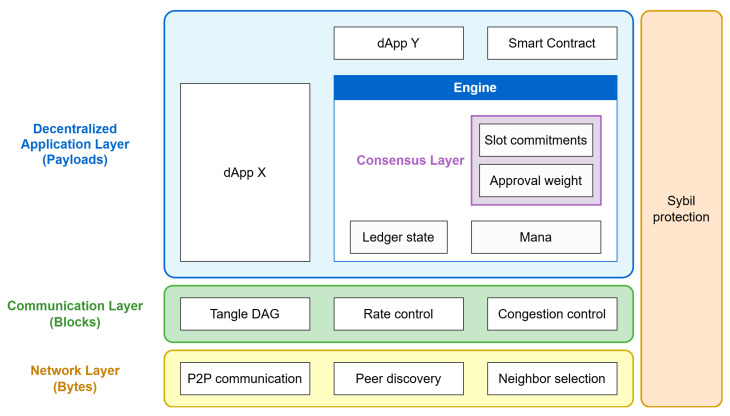
Layered architecture of the IOTA 2.0 protocol [29].

IOTA 2.0 adopted a modular, layered architecture comprising three main layers—network, communication, and application—each building upon the functionalities of the one beneath it. At the foundational network layer, a peer-to-peer (P2P) overlay facilitates secure and efficient peer discovery and neighbor selection through mechanisms like autopeering, allowing nodes to connect automatically. This layer also manages message propagation via a gossip protocol to maintain network connectivity. Built upon this is the communication layer, responsible for the dissemination and processing of blocks that form the Tangle DAG structure. It employs rate control and congestion control modules to regulate data flow and prevent network congestion. Finally, at the highest level, the decentralized application layer encompasses core ledger functionalities, including the ledger engine responsible for maintaining the state of the ledger and resource management through Mana. Additionally, this layer supports decentralized applications (dApps) and smart contracts, enabling developers to build and deploy various services directly onto the IOTA platform [29]. Figure 3 presents the layered architecture of the IOTA 2.0 protocol.

### 3.3. Unmet Goals and Challenges of IOTA 2.0

Despite its pioneering vision, IOTA 2.0 encountered unresolved challenges that impeded its deployment. This subsection examines critical hurdles, including consensus and finalization complexities and prolonged development cycles.

#### 3.3.1. Consensus Mechanism Complexities

The FPC mechanism and Nakamoto Consensus implemented in IOTA 2.0 represented a substantial improvement over the coordinator-based system. However, it introduced complexity that posed challenges to seamless network operation. Under challenging network conditions—such as network partitions or when validators go offline—nodes may perceive different blocks being accepted, creating divergent slot commitment chains. This can lead to network forks where different nodes maintain inconsistent views of the ledger state. While IOTA 2.0 implements a Chain Switching Rule to resolve these inconsistencies, the very existence of temporary forks introduces uncertainty and operational complexity. The protocol requires nodes to track and update their slot commitment chains while checking commitments from other nodes, adding significant computational overhead [30].

#### 3.3.2. Finalization Process Complexity

The finalization process in IOTA 2.0 required a supermajority (more than two-thirds of voting power) of the validators committee to reference slot commitments in their validation blocks. All validation blocks had to be issued within the same slot and be confirmed to achieve finalization. Each node computed finalization flags based on its own locally maintained copy of the Tangle [30]. This rigorous finalization process enhanced security but introduced latency that might have impacted time-sensitive applications. The complexity of this mechanism creates potential barriers to adoption for developers unfamiliar with the unique consensus approach of IOTA 2.0.

#### 3.3.3. Protracted Development Cycle

One of the most significant challenges limiting the adoption of IOTA 2.0 has been its extended development timeline. The journey toward a fully decentralized IOTA began years ago, with initial testing network deployments occurring as early as 2020. The development progressed through multiple stages, beginning with the Pollen testing network (the first testing phase of IOTA 2.0), then advancing to the Nectar test network (a full implementation of IOTA 2.0 on an incentivized testing network), with the final aim of implementing the Honey main network (including all modules and final specifications of the Coordicide) [31]. However, as of early 2024, the full implementation of IOTA 2.0 appeared to remain a work in progress, with the IOTA Foundation still making progress in the research and development of IOTA 2.0. By the end of 2024, the IOTA 2.0 project had finally been abandoned after 7 years of continuous development, and the ledger switched to IOTA Rebased following a vote by IOTA token holders, with the IOTA Foundation acknowledging that this switch would make time-to-market for full decentralization shorter than IOTA 2.0 plans [32,33]. This extended development cycle of the IOTA 2.0 protocol has dampened enthusiasm among potential adopters and developers, potentially leading to exploring other blockchain solutions with more stable and established protocols during that period.

#### 3.3.4. Architectural Refinements and Optimizations

The IOTA Foundation has acknowledged the need for substantial protocol refinements, indicating that the initial design may have been suboptimal. Continuous modifications suggested that the original architecture may have contained inefficiencies or unnecessary complexity that required remediation. In September 2021, the foundation reported working on optimizing the consensus layer along with the communication layer to enhance protocol efficiency and reduce its complexity, with the majority of changes involving optimization of the data flow and code simplification [34]. Such iterative refinements, while necessary for protocol improvement, have extended the timeline for a production-ready implementation and created uncertainty around the final form and capabilities of IOTA 2.0.

#### 3.3.5. Smart Contracts Functionality Implementation

IOTA 2.0 was designed to support smart contracts, addressing a significant limitation of the original protocol. However, the implementation of this functionality faced delays due to the unique architecture of the IOTA 2.0. The introduction of the Shimmer EVM followed by the IOTA EVM launch suggested that the original smart contract design may not have met market requirements, raising concerns regarding the scalability, security and user-adaptability of the protocol design. While the integration of the Sui MoveVM—a novel smart contract language for programming blockchains using objects abstraction [35]—was considered feasible by the IOTA Foundation, its implementation in IOTA 2.0 architecture would have been a multi-year effort that could have caused additional undesirable delays due to breaking protocol changes [33]. Commendable progress was made in ledger performance, but the necessity of implementing fundamental revisions for Layer 1 programmability in IOTA 2.0 raised pertinent questions about the overall stability and developmental maturity of the protocol, with the IOTA Foundation recognizing that it could not afford additional years of research and development with uncertain outcomes [32].

#### 3.3.6. Competition from Other Blockchains

IOTA 2.0 entered a highly competitive landscape dominated by established smart contract platforms like Ethereum and rapidly growing alternatives such as Solana and Sui [36,37]. These platforms offer mature developer ecosystems, extensive tooling, and proven scalability that have attracted significant developer communities and user bases. Continuous design revisions in IOTA 2.0 made it challenging to compete with other well-established Layer 1 blockchains that offered consistent performance metrics.

#### 3.3.7. Hardware and Resource Constraints

The IOTA design philosophy of maintaining low hardware requirements to accommodate unpaid node operators created a fundamental tension with performance objectives. While this approach aligns with the initial IOTA vision of enabling IoT applications on resource-constrained devices, it simultaneously limits the ability of the protocol to scale to meet competitive performance benchmarks. This trade-off may be particularly problematic for attracting institutional users and developers building high-throughput applications who prioritize performance over hardware efficiency.

## 4. IOTA Rebased: A Technical Overview

### 4.1. Technical Foundation

IOTA Rebased reimagines the architecture of the protocol to prioritize scalability and programmability. This subsection details its technical pillars: an object-oriented DAG ledger for parallel transaction processing, Mysticeti consensus for Byzantine fault tolerance with sub-second finality, and MoveVM-based smart contracts for enhanced programmability.

#### 4.1.1. Object-Based DAG Ledger

IOTA Rebased fundamentally restructures the previous Tangle implementation by transitioning from the UTXO model to an object-based DAG ledger inspired by the Sui Network. This enables enhanced programmability and parallel transaction processing and significantly enhances throughput capabilities. The object model in IOTA Rebased differentiates between shared and owned objects. Shared objects represent resources that multiple entities can access and modify according to predefined rules, while owned objects are exclusively controlled by their designated owners. This distinction facilitates fine-grained access control and enables more complex transaction patterns within the network [9,33].

#### 4.1.2. Mysticeti Consensus Protocol

IOTA Rebased integrates the Mysticeti protocol [10], a BFT consensus protocol optimized for low latency and high throughput using an uncertified DAG structure. Delegated Proof of Stake (DPoS) is used as a consensus mechanism in which token holders delegate their voting power to validators who participate in block production and finalize transactions by executing the Mysticeti protocol [38]. Mysticeti solves the high latency issue in certified DAGs by eliminating the need for explicit certificates, achieving block commitment within the known lower bound of 3 message rounds. Furthermore, it enhances efficiency by committing each block independently without waiting for the entire wave to conclude and minimizes CPU overhead by requiring only a single signature generation and verification per block.

The Mysticeti protocol is proposed in two classes: Mysticeti-C, which is the main protocol version, and Mysticeti-FPC, which adopts a fast commit path that lowers the latency in asset transfer. Both Mysticeti protocol classes reach decisions by interpreting the structure of the DAG, utilizing only block messages. Mysticeti protocol classes operate in a sequence of logical *rounds*. Honest validators propose a unique signed block each round, incorporating transactions and references to recent blocks from prior rounds. A block is signed and sent once it references at least 2f+1 blocks from the previous round, with *f* being the number of validators. Clients submit transactions to validators; if a transaction is not finalized within a specific time frame, the client resubmits it to a different validator. Byzantine validators might attempt to equivocate by sending multiple or no blocks to different parties. Such validators are neutralized through DAG pattern analysis, akin to fault predictability in labeled Petri nets [39]. Whereas Petri nets use transition invariants to anticipate faults, Mysticeti dynamically identifies adversarial blocks by requiring validators to reference prior rounds’ certificates. This structural approach ensures Byzantine resilience without relying on static fault models, aligning with the unpredictable nature of decentralized networks.

For a block to be correct, it must include the author *A* and signature on the block contents, a round number *r*, a list of transactions, and at least 2f+1 hashes *h* of blocks from the previous round (plus potentially other previous rounds), with the first hash linking to the previous block of *A*. A block B≡(A,r,h) is valid if the signature is valid, *A* is a validator, all hashes point to distinct valid blocks from previous rounds, the first block links to another block from *A*, and the sequence of past blocks contains 2f+1 blocks from the immediate past round r−1. A block $B^{\prime }$ supports a past block B≡(A,r,h) if, when tracing back from $B^{\prime }$ through hashed blocks, *B* is the first block found for validator *A* at round *r*. While a block might reference others (from different validators) that support equivocating blocks at the same round, at most one of these equivocating blocks can receive support from 2f+1 validators.

**Figure 4:**
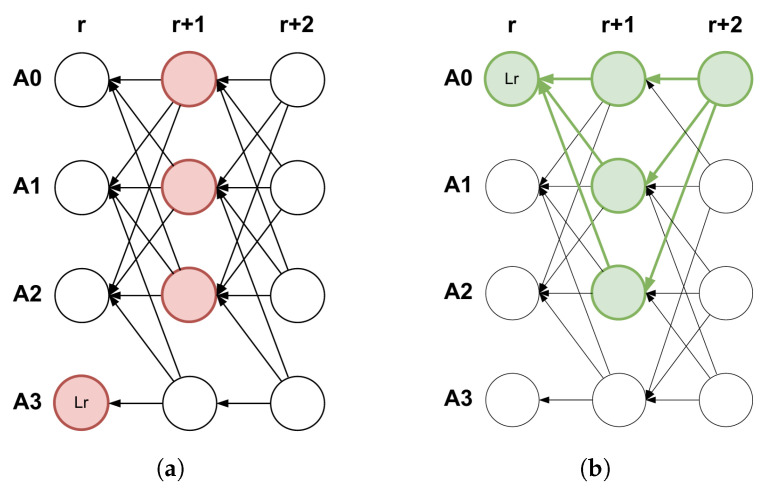
The main DAG patterns identified by validators in the Mysticeti protocol [10]. (**a**) In this skip pattern (formed by red blocks), blocks $\left(A_{0},r+1,\cdot \right),\left(A_{1},r+1,\cdot \right),\left(A_{2},r+1,\cdot \right)$ do not support $\left(A_{3},r,L_{r}\right)$. (**b**) In this certificate pattern (formed by green blocks), block $\left(A_{0},r+2,\cdot \right)$ serves as a certificate for $\left(A_{0},r,L\right)$.

Mysticeti protocol classes operate primarily by involving the identification of two main patterns by validators:

- The skip pattern (Figure 4a) is identified at round r+1 when at least 2f+1 blocks do not support a specific block (A,r,h). Regardless of the number of proposals for the slot (including none), the skip pattern is identified if, across all proposals, we observe 2f+1 subsequent blocks that either do not support it or support no proposal at all.
- The certificate pattern (Figure 4b) is identified at round r+1 when at least 2f+1 blocks support a block B≡(A,r,h). In this case, block *B* is considered certified, guaranteeing its availability and that no other certified block may exist for the same spot (A,r). Any subsequent block (like one at round r+2) that includes this pattern within its history is called a certificate for block *B*.

In IOTA Rebased, Mysticeti is used with a validator committee of initially 150 permissionless slots, selected through staking by token holders. Validators collectively determine transaction validity and finality in 3 message rounds, ensuring economic alignment between network security and stakeholder interests [9,33]. This new consensus scheme in IOTA is designed for fast finality to reduce the risk of rollbacks, energy efficiency compared with the PoW, and parallel transaction processing while maintaining strong security guarantees [38]. The introduction of a leader-based DAG-BFT mechanism allows validators to propose blocks asynchronously, making IOTA Rebased achieve 0.5-s finality with more than 50,000 TPS with sub-second finality [33].

The IOTA Foundation reported significant progress on the development of an evolution of Mysticeti, named the *Starfish* protocol, a partially synchronous DAG-based BFT protocol that incorporates the security properties of certified DAGs, the efficiency of uncertified DAG approaches, and linear amortized communication complexity. The key innovation in Starfish is the Encoded Cordial Dissemination (ECD), a push-based dissemination strategy that combines Reed-Solomon erasure coding with Data Availability Certificates (DACs). This lowers communication costs per byte, ensuring suitability for large-scale DLT deployments. Starfish experimental results demonstrated improved robustness and stable latency over Mysticeti, especially under steady-state and Byzantine scenarios, along with potentially higher throughput reportedly up to 150,000 TPS. The Starfish upgrade will include a fairer top-stakers committee selection process that allows higher-stake validators to replace underperforming ones [40].

#### 4.1.3. Transactions Life Cycle

**Figure 5:**
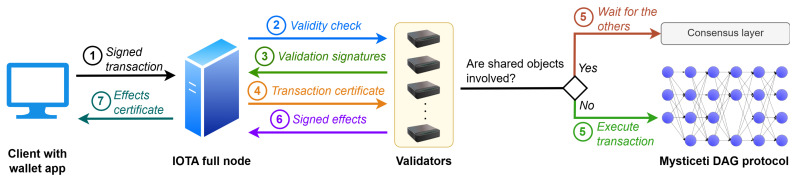
Transactions life cycle in IOTA Rebased.

The transaction life cycle in IOTA Rebased is designed as follows. Firstly, the transaction is created by a client, whose wallet signs the transaction with the private key and submits the signed transaction to an IOTA full node for processing. The full node, not having the full view of all transactions within the network, broadcasts the transaction to all validators, who check its validity, sign it in the case it is valid, and return the signature to the full node after having locked the involved client-owned objects to prevent double-spending. The full node collects signatures in parallel from a supermajority (quorum) of validators to certify the transaction and establish its certificate. Once certified, transactions are sent to validators for execution, who first verify the certificate signatures to confirm the validity of the transaction and prevent double-spending. If no shared objects are involved, validators will instantly execute the transaction (fast path consensus); otherwise, they will submit it to the consensus layer to order the transaction with others sharing the same object and then execute it. After a transaction, validators sign its *effects* (details about objects involved, amount spent, and transaction status) and send them back to the full node. Once the full node receives signed effects from a majority of validators, it creates an *effects certificate*, guaranteeing that the transaction is final and will be included in a future checkpoint—the final stage in the life of that transaction. Validators submit executed transactions to the consensus layer for universal ordering, and checkpoints are then created using this ordered list of transactions and their corresponding effects [41]. Figure 5 summarizes transaction life cycle in IOTA Rebased.

#### 4.1.4. Smart Contracts Virtualization

In this object-based design, each transaction creates, modifies, or deletes objects, which are stateful entities representing assets, smart contracts, or metadata. This enables Layer 1 programmability and simplifies complex use cases like supply chain tokenization. Resource-oriented programming is enabled as the MoveVM resource model ensures type safety and eliminates reentrancy vulnerabilities, critical for financial applications. Developers define custom assets (e.g., tokens, NFTs) as immutable resources, enforced at the protocol level.

The Rebased architecture supports dual virtualization: Layer 1 smart contracts powered by the MoveVM and Layer 2 IOTA EVM compatibility. The MoveVM provides static verification, formal validation of contract logic, and robust ownership guarantees through its linear type system, thereby enhancing security and simplifying development for financial instruments and other complex applications. The integration of IOTA EVM as a parallel execution environment allows developers to port Solidity-based dApps while leveraging the scalability of IOTA. This EVM compatibility at Layer 2 ensures seamless migration for existing Ethereum-based dApps, broadening ecosystem appeal while supporting diverse development needs. A future *MultiVM* upgrade aims to unify MoveVM and IOTA EVM under a single Layer 1 security model [9].

### 4.2. Tokenomics

IOTA Rebased introduces a revised economic model to sustain network operations and incentivize participation. This subsection examines its fee-driven structure with adaptive gas pricing, staking rewards for validators and delegators, and controlled inflation to balance token supply.

#### 4.2.1. Fee Model

IOTA Rebased introduces an initial *gas fee* of about 0.005 IOTA per average transaction, with an adaptive fee burn mechanism to balance inflationary and deflationary dynamics based on network activity. Storage deposits (refundable) are also required for on-chain data, discouraging spam. To mitigate IoT friction, developers and businesses can subsidize fees for users via the IOTA Gas Station, a tool allowing dApps to cover user costs. This preserves accessibility for microtransactions through sponsored transactions [42].

#### 4.2.2. Stacking and Inflation

The tokenomics model in IOTA Rebased is designed to incentivize network participation and ensure long-term sustainability. Validators, who secure the network, will be rewarded with newly minted tokens in the form of staking rewards. Delegators share rewards proportionally to incentivize participation. The initial annual inflation rate is estimated to be 6% in the first year and then gradually decline to a constant rate, with burnt fees (0.005 IOTA per transaction) offsetting inflation [9].

Overall, the revised tokenomics are designed to provide a self-sustaining economic model that supports validator operations and network maintenance while offering a clear incentive structure for participants. The fee-based model, combined with staking and dynamic validator committees, introduces a robust economic deterrent against attacks. The cost associated with misbehavior or malicious activities is increased, thereby enhancing overall security.

## 5. Comparative Analysis of IOTA 2.0 with Rebased

### 5.1. Consensus Mechanism

Nakamoto Consensus on a DAG in IOTA 2.0 is designed to maximize decentralization by involving all network nodes in validation. Conversely, the Mysticeti DPoS-based model in Rebased sacrifices some of this decentralization in favor of higher processing speed and scalability. While the first consensus mechanism prioritizes decentralization, the latter puts network efficiency at the forefront.

In IOTA 2.0, consensus is gradually achieved as more transactions add weight to valid blocks, which may result in longer confirmation times. The Mysticeti approach in Rebased, however, offers near-instant finality through its validator committee, making it more suitable for applications that require rapid transaction processing. While the first focuses on finality, the latter prioritizes network throughput.

While both consensus mechanisms strive to secure the network, Nakamoto Consensus on a DAG depends on the organic growth of cumulative weight to prevent double-spending. In contrast, Mysticeti utilizes a validator committee that can efficiently manage Byzantine faults, with potential vulnerabilities if the committee becomes unbalanced or subject to collusion.

Overall, Nakamoto Consensus on a DAG in IOTA 2.0 emphasized community-driven, fully decentralized validation at the cost of complexity, whereas Mysticeti consensus in IOTA Rebased focuses on achieving high throughput and low latency in a more secure consensus, albeit with a somewhat more centralized validator model.

### 5.2. Transaction Economics

One of the cornerstone promises of IOTA 2.0 was the ability to conduct feeless microtransactions, with the Mana system preventing network spamming without monetary costs. This design was particularly attractive for IoT devices, where high-frequency, low-value transactions are common. IOTA Rebased, on the other hand, introduces minimal transaction fees based on a gas pricing model. These fees serve as a mechanism to discourage spam, incentivize validators and delegators, and maintain the economic integrity of the network. This transition from feeless to fee-based transactions represents a significant departure from the original IoT-friendly vision, undermining IoT usability. Although fees help create a sustainable economic model and improve network security, they also risk limiting the utility of IOTA for high-volume, low-cost IoT applications in the Big Data era.

### 5.3. Scalability

IOTA 2.0 was designed to allow parallel processing of transactions and enable the network to scale naturally with increased activity, as more transactions contributed to the validation process. However, while this approach promoted decentralization and resilience, it may have experienced variable confirmation times, especially under high load, as the system relied on the accumulation of approval weight to finalize transactions.

On the other hand, the Mysticeti protocol with DPoS in IOTA Rebased, along with a committee of initially 150 permissionless validators processing transactions, achieves high throughput—reportedly over 50,000 TPS—with sub-second finality. This structure allows for rapid transaction processing and is well-suited for applications requiring high-speed confirmations. However, the reliance on a fixed validator set introduces a degree of centralization, which could impact network adaptability and resilience.

Overall, IOTA Rebased offers superior performance metrics suitable for applications demanding high-speed transactions, while IOTA 2.0 provides a more decentralized and adaptable framework that scales with network activity.

### 5.4. Security Considerations

IOTA 2.0 sought to enhance security by distributing the validation process across all network participants and using Mana as a reputation system to mitigate Sybil attacks. The removal of the centralized coordinator was intended to eliminate single points of failure in the network. With IOTA Rebased, security is enforced through economic incentives. Validators are required to stake tokens, and misbehavior carries financial penalties. The periodic reconfiguration of validator committees further secures the network against long-term attacks.

The two models represent different approaches to achieving security. IOTA 2.0 focused on distributed trust and reputation, whereas IOTA Rebased relies on economic disincentives and structured validator selection. Although the staking mechanism in IOTA Rebased enhances economic security, it also introduces new challenges in ensuring equitable participation across diverse IoT devices.

### 5.5. Implications for IoT Applications

The feeless model of IOTA 2.0 was ideally suited for the high-frequency, low-value transactions typical in IoT environments. The reintroduction of fees in IOTA Rebased may complicate this scenario, as even minimal fees can accumulate and affect the cost-efficiency of microtransactions in the case of large amounts of data. While IOTA 2.0 encouraged broad participation through autonomous validation, IOTA Rebased requires IoT devices or their proxies to engage in staking and validation processes. This may impose additional resource requirements on devices not originally designed for such tasks. Although the optimized consensus provided by the Mysticeti protocol in Rebased offers improved transaction finality and network adaptability, these benefits must be weighed against potential increases in operational complexity and the economic barriers introduced by transaction fees in specific cases.

Table 2 summarizes the advantages and drawbacks of the IOTA Rebased protocol with respect to the previously proposed IOTA 2.0 protocol.

## 6. Challenges and Recommendations for Adopting IOTA Rebased in IoT

### 6.1. Key Challenges

Adopting IOTA Rebased in IoT ecosystems introduces critical challenges that conflict with its original mission of enabling frictionless machine-to-machine transactions. This subsection identifies barriers such as transaction fees undermining microtransactions, staking requirements incompatible with resource-constrained devices, and technical complexity in bridging the object-oriented model of Rebased with legacy IoT protocols.

#### 6.1.1. Reintroduction of Transaction Fees

The feeless design of IOTA 2.0 was specifically tailored for the high-frequency, low-value transactions common in IoT ecosystems. With IOTA Rebased introducing even minimal fees, the economic viability of microtransactions may be compromised, especially in the context of Big Data with millions of IoT devices making billions of transactions. Even low fees could accumulate when millions of IoT devices are transacting, potentially deterring their use in real-time applications and breaking the business case for millions of tiny telemetry messages. Additionally, IoT devices often operate with very tight energy and computational budgets. Adding fee calculations and fee-burning mechanisms could increase overhead, complicating the seamless integration of devices with the network.

#### 6.1.2. Staking and Validator Participation

Many IoT devices are resource-constrained and may lack the capacity to participate directly in staking or validation processes. This forces reliance on proxy nodes or gateway solutions, which can introduce additional points of failure and potential centralization risks. In addition, the DPoS model and staking requirements may favor participants with substantial token holdings and robust infrastructure. This could potentially lead to a concentration of network control among a few entities, counteracting the original decentralization promise of IOTA.

Hardware requirements for validators in IOTA Rebased have been initially set to 24-core CPUs, 128 GB RAM, 4 TB NVMe, and 1 Gbps uplink, plus a 2M IOTA stake per validator [43]. Such hardware is orders of magnitude beyond what typical IoT gateways can host, forcing edge operators to rely on remote data center nodes. This limitation hinders the possibility of IoT devices to participate in network throughput and scaling by acting as validators in a fully decentralized leaderless DLT.

#### 6.1.3. Technical Complexity and Integration

Integrating Move-based smart contracts directly on Layer 1 adds a level of complexity in the context of IoT. While Move offers enhanced security and programmability, its adoption requires a steep learning curve and robust developer support. In an IoT context, where seamless interoperability is key, ensuring that legacy IoT devices and software can communicate with the new protocol poses a noticeable challenge.

#### 6.1.4. Security and Network Scalability

While staking and fee-burning mechanisms bolster economic security, they can also deter smaller participants around the world from joining the network. Ensuring robust security without sacrificing the global inclusive goal of DLTs for IoT networks is a delicate balance.

The use of Mysticeti consensus in replacement of Nakamoto on DAG must be rigorously tested under IoT-scale high transaction loads, since the network throughput no longer scales with network activity. While IOTA Rebased promises high scalability (over 50,000 TPS), large-scale IoT deployments may involve millions of devices generating transactions. The transition from a DAG-based architecture to an object-based ledger with a DPoS consensus mechanism raises questions about scalability under extreme conditions. Any latency or stability issues in high-load scenarios could impede the real-time communication essential for large-scale, high-load IoT deployments.

### 6.2. Strategic Recommendations

To mitigate the challenges of adopting IOTA Rebased in IoT environments, this subsection proposes actionable strategies. While the proposed approaches aim to align Rebased with IoT demands, they necessitate trade-offs between decentralization, efficiency, and economic viability.

#### 6.2.1. Subsidization and Tiered Fee Structures

Sponsored transactions can help preserve accessibility for microtransactions, as well as other models where fees for IoT microtransactions are partially or fully subsidized by network incentives or partnerships [44]. This approach could help preserve the low-cost transaction benefits that are critical for IoT use cases. In addition, designing a fee structure that adjusts based on transaction size or frequency can help ensure that low-value transactions incur negligible costs while maintaining the economic balance of the network. Still, while the new IOTA Gas Station lets integrators sponsor fees, it shifts operational expenses to application owners instead of the protocol.

#### 6.2.2. Lightweight Client Protocols and Proxies

To address resource constraints in IoT devices, specialized lightweight client implementations can allow resource-constrained IoT devices to interact with the IOTA Rebased network indirectly, through proxies [45]. In such hierarchical tiered transaction architecture, these proxies would handle staking on behalf of multiple resource-constrained IoT devices, reducing the burden on individual IoT nodes, but at the cost of more centralization. However, attention should be paid to implementing redundancy in order to avoid these proxies becoming single points of failure. Efficient caching mechanisms should be considered to minimize redundant computations for repetitive transactions common in IoT applications.

#### 6.2.3. Batch Transactions

The batch transactions paradigm [46,47,48] can be implemented on a case-by-case basis to minimize fee overhead for frequent low-value transactions in IoT deployments where real-time data transfers are not required. This could be combined with fee estimation frameworks, creating predictive models that help IoT systems estimate the necessary budget for transaction fees in various network conditions. This implies reevaluating how value is captured in IoT applications to shift from many small transactions to fewer higher-value aggregated transactions.

#### 6.2.4. Adaptive Transaction Scheduling

In a hierarchical transaction architecture, intelligent adaptive transaction scheduling can be implemented to prioritize time-sensitive operations while delaying non-critical transactions in the case of network congestion [45,49]. This strategy can be combined with data processing and aggregation at the tier edge to reduce the volume of transactions that need to be recorded on the main ledger. Value-based transaction filtering algorithms on the tier edge can help determine which data are valuable enough to warrant on-chain storage versus what can be processed off-chain.

### 6.3. Use Cases Analysis

This subsection evaluates the applicability of IOTA Rebased through real-world IoT scenarios, including smart energy grids, supply chain tracking, and healthcare monitoring. These use cases underscore the potential of Rebased in enterprise-scale applications, albeit with adaptations to preserve its original vision for decentralized machine economies.

#### 6.3.1. Smart Energy Grids and Peer-to-Peer Energy Trading

Energy microgrids typically involve numerous small energy transactions between prosumers (producer-consumers). IOTA Tangle has been explored for secure and scalable P2P energy trading [50,51,52]. The introduction of fees in IOTA Rebased could make very small energy trades uneconomical. To overcome this constraint, one could aggregate energy transactions over time periods (e.g., hourly or daily) rather than recording each kilowatt-hour exchange. Sponsored transactions can be implemented, where energy service providers cover transaction fees as part of their service offering. In addition, staking rewards from a community energy pool can be used to offset transaction costs.

#### 6.3.2. Supply Chain Tracking and Provenance

Supply chains involve multiple stakeholders and numerous tracking events. Several works in the literature explored the use of IOTA Tangle in supply chain tracking and provenance certification [53,54]. Adding fees to each tracking update in IOTA Rebased could increase operational costs. An efficient implementation strategy could include selective on-chain recording, where only critical handoffs and quality measurements are recorded on-chain. Batch updates can be used for non-critical tracking events, further reducing operational expenses. In addition, a cost-sharing model can be implemented where each supply chain participant stakes IOTA tokens and shares in both staking rewards and transaction fees.

#### 6.3.3. Vehicle Communication Systems

Vehicle-to-everything communications often require real-time, high-frequency data exchanges that may become prohibitively expensive if each message incurs a fee. The IOTA Tangle was proposed for securing V2X communications in scalable decentralized networks [55,56]. With the fee-based model of IOTA Rebased, a tiered data model can be implemented, where safety-critical messages use direct on-chain transactions with sponsored fees. Off-chain channels can be utilized for routine telemetry data with periodic checkpoints. Additionally, to encourage participation, a token economics model can be developed, where vehicles earn tokens by providing valuable road condition data that offsets the costs of their transactions.

#### 6.3.4. Healthcare Remote Monitoring

Continuous patient monitoring generates high volumes of data, most of which is routine but occasionally includes critical alerts. IOTA Tangle demonstrated suitability in the context of secure healthcare data management and sharing [57,58]. In IOTA rebased, with the introduction of fees, one could implement smart filtering algorithms that only record clinically significant changes on-chain. Healthcare providers or insurance companies can be involved to cover transaction sponsoring on IOTA Rebased. Further design can implement value-based data models where the importance of the data determines its recording strategy.

## 7. Potential Research Directions

The evolution of IOTA from a feeless, IoT-centric DAG to a fee-driven, object-oriented ledger necessitates targeted research to reconcile its original vision with modern scalability and security demands. Below, we outline key directions to advance the applicability of the IOTA in IoT ecosystems.

### 7.1. Adaptive Indexing and QUERY Optimization for DAGs

The branching structure of the IOTA DAG makes efficient hierarchical data retrieval more complex than in linear blockchains, particularly for resource-constrained IoT devices. Future work should explore machine learning-driven indexing techniques, such as graph neural networks trained on transaction metadata, to predict transaction locations dynamically. However, concurrent branches in the IOTA DAG necessitate dynamic index updates, posing challenges for machine learning model accuracy. Hybrid approaches combining learned models with heuristic DAG traversal could mitigate the computational overhead of real-time updates. Adapting blockchain-optimized frameworks like Telex—a two-level learned index [59]—to the IOTA DAG could further enhance query efficiency, though challenges remain in balancing model accuracy with the concurrent validation processes in IOTA.

### 7.2. Lightweight Privacy-Preserving Mechanisms

Enabling encrypted rich queries on IOTA without compromising IoT device efficiency requires novel privacy-preserving techniques. Oblivious RAM (ORAM) protocols, paired with Trusted Execution Environments like Intel SGX or ARM TrustZone, could obfuscate access patterns while ensuring forward and backward privacy in the DLT [60,61]. Simplified ORAM variants optimized for edge devices, coupled with lattice-based cryptography, may reduce computational overhead. Such mechanisms would allow IoT devices to securely query ledger data (e.g., supply chain events) without exposing sensitive access patterns.

### 7.3. Scalability Under Extreme IoT Loads

Validating the performance of IOTA Rebased at IoT scale with millions of devices generating thousands of transactions per second requires stress-testing frameworks. Sharded validator committees and adaptive consensus protocols (e.g., dynamically adjusting block sizes in Mysticeti) could enhance throughput. Simulation tools modeling high-density IoT deployments such as smart cities would identify bottlenecks in transaction finality and network resilience. Recently, reinforcement learning-based approaches have proven to improve decentralization and efficiency of DAG-based DLTs, ensuring stable scalability and high security resilience [62].

### 7.4. Energy-Efficient Participation

Resource-constrained IoT devices struggle with staking and validation tasks. Delegated validation mechanisms, where edge gateways act on behalf of low-power devices, could democratize participation. Energy-efficient consensus variants, such as proof-of-uptime [63], might further optimize computational demands while maintaining security.

### 7.5. Convergence with Other Emerging Technologies

Synergies with 5G, 6G, edge AI, and federated learning present opportunities to optimize IOTA for next-generation IoT. Federated learning frameworks could leverage IOTA for secure model aggregation across edge devices [64,65], while the ultra-low latency in 5G and 6G networks might enhance sub-second finality of Mysticeti in vehicular networks.

## 8. Conclusions

The transition from IOTA Tangle 2.0 to IOTA Rebased marks a pivotal moment in DLTs, reflecting broader tensions between ideological purity and pragmatic evolution. While IOTA 2.0 pioneered a feeless, leaderless DAG architecture tailored for the IoT, its complexity and delayed smart contract functionality hindered its adoption. IOTA Rebased, leveraging MoveVM and Mysticeti DAG protocol, prioritizes enterprise scalability, sub-second finality, and cross-chain interoperability, but at the cost of abandoning the IOTA foundational promise of feeless transactions and secure data exchange.

Our comparative analysis reveals that this evolution introduces both opportunities and challenges, particularly in reconciling the original IoT-friendly vision with the practical demands of maintaining a secure, scalable, and economically viable distributed ledger. Mitigating these issues will demand, among other recommendations of this paper, a tiered architecture where edge gateways speak for sensor swarms, along with fee-sponsorship models. Future research and development will need to address these trade-offs to ensure that IOTA continues to serve as a robust platform for machine-to-machine communication in an increasingly connected world.

## Figures and Tables

**Table 1:** Protocol versions of the IOTA Tangle [13].

Protocol	Release Date	Description	Main Features
IOTA 1.0	July 2016	The legacy version of the IOTA Tangle, using a probabilistic consensus algorithm based random walkers to select most suitable tips for approval.	Trinary data representation.
Chrysalis 1 (IOTA 1.5)	August 2020	The White-Flag approach replaces the heavy probabilistic consensus algorithm, addressing the conflict spamming attack by enforcing a deterministic ordering of the Tangle.	White-Flag confirmation.
Chrysalis 2 (IOTA 1.5)	April 2021	Transitioned from trinary to binary data representation, replaced the account model with the UTXO model, migrated from Winternitz One-Time Signature to EdDSA signature scheme, and implemented measures to guard against dust transactions.	Binary data representation, UTXO, EdDSA.
Stardust	October 2023	Introduced support for multiple assets support, implemented byte cost-based granular dust protection through storage deposits, evolved vertices from messages to blocks, and prepared for ISC anchoring via Alias Outputs, replacement of the single Coordinator node with a validation committee.	Multiple assets, smart contracts, byte cost-based dust protection, blocks as vertices, Bench32 addresses, validation committee.
IOTA Rebased	May 2025	Reimagined ledger architecture replacing the Tangle with the Mysticeti DAG, introducing transaction fees, MoveVM-based smart contracts, and a Delegated Proof-of-Stake (DPoS) consensus model via the Mysticeti protocol.	Object-oriented ledger, Mysticeti protocol, transaction fees, MoveVM integration, DPoS consensus.

**Table 2:** Comparison of IOTA Rebased with the proposed IOTA 2.0.

Feature	IOTA 2.0	IOTA Rebased
Ledger model	UTXO-based Tangle DAG with simpler microtransaction logic	Object-oriented ledger enabling Layer 1 smart contracts at the cost of microtransaction logic
Hardware requirements	Light nodes could operate on low-power IoT devices	MoveVM and Mysticeti demand higher computational resources, limiting edge devices participation
Consensus	Nakamoto on DAG, prioritizes decentralization	DPoS with Mysticeti, high throughput and low latency, 0.5-s finality
Transaction fees	Feeless, ideal for microtransactions	Minimal fees, supports economic sustainability
Scalability	Theoretically high TPS, scaling with network activity, potentially variable confirmation times	Over 50,000 TPS, fixed set of validators, suited for high-speed transactions
Security	Decentralized validation with Mana for reputation	Economic security via staking and periodic validator refresh
Smart contracts	Layer 2 with limited Layer 1 support	Native Layer 1 with Layer 2 solutions, MoveVM and IOTA EVM, supports complex dApps
Governance	Permissionless, decentralized, fully inclusive	On-chain voting, DPoS validators committee, faster but less inclusive
IoT suitability	Optimized for low-cost, high-frequency transactions	Economic model may hinder low-value transactions in IoT setups

## Data Availability

Not applicable.

## Abbreviations

The following abbreviations are used in this manuscript:

IoT	Internet of Things
DLTs	Distributed Ledger Technologies
DAG	Directed Acyclic Graph
PoW	Proof-of-Work
PoA	Proof-of-Authority
UTXO	Unspent Transaction Output
EdDSA	Edwards-curve Digital Signature Algorithm
ISC	IOTA Smart Contracts
BFT	Byzantine Fault Tolerant
EVM	Ethereum Virtual Machine
TPS	Transactions Per Second
NFTs	Non-Fungible Tokens
MEV	Maximal Extractable Value
FCOB	Fast Confirmation of On-the-Heavy Branch
FPC	Fast Probabilistic Consensus
dRNG	distributed Random Number Generation
BIC	Block Issuance Credits
DoS	Denial of Service
CPS	Cyber-Physical Systems
P2P	Peer-to-Peer
dApps	Decentralized Applications
DPoS	Delegated Proof-of-Stake
ECD	Encoded Cordial Dissemination
DAC	Data Availability Certificate
ORAM	Oblivious RAM
